# Inequities in air pollution on stroke mortality among Older Americans: a U.S. nationwide analysis

**DOI:** 10.3389/fpubh.2024.1364165

**Published:** 2024-09-23

**Authors:** Jean-Luc K. Kabangu, Danny Fowler, Amanda Hernandez, Takara Newsome-Cuby, Benson Joseph, John Dugan, Lane Fry, Momodou G. Bah, Sonia V. Eden

**Affiliations:** ^1^Department of Neurological Surgery, University of Kansas Medical Center, Kansas City, KS, United States; ^2^New York Institute of Technology, College of Osteopathic Medicine at Arkansas State University, Jonesboro, AR, United States; ^3^University of Michigan Medical School, Ann Arbor, MI, United States; ^4^College of Osteopathic Medicine, Kansas City University, Kansas City, MO, United States; ^5^Department of Surgery, University of Tennessee Health Science Center, Memphis, TN, United States; ^6^University of Tennessee Health Science Center College of Medicine, Memphis, TN, United States; ^7^University of Kansas School of Medicine, Kansas City, KS, United States; ^8^Michigan State University College of Human Medicine, East Lansing, MI, United States; ^9^Department of Neurosurgery, Semmes-Murphey Clinic, Memphis, TN, United States; ^10^University of Tennessee Health Sciences Center, Memphis, TN, United States

**Keywords:** PM2.5, stroke mortality, health disparities, air pollution, health equality

## Abstract

**Background:**

Air pollution is a known risk factor for cardiovascular diseases, including stroke. This study examines the impact of county-level air pollution on ischemic and hemorrhagic stroke mortality among U.S. individuals aged 65 and older, emphasizing racial and socioeconomic disparities.

**Methods:**

Using data from the Center for Disease Control (CDC) Interactive Atlas of Heart Disease and Stroke, we analyzed county-level ischemic stroke mortality rates for older residents between 2016 and 2020. The data on air pollution at the county level, specifically particulate matter (PM2.5) levels, were obtained from the CDC. We applied multivariable linear and logistic regression models to examine the association between PM2.5 levels and stroke mortality, as well as the probability of meeting the Environmental Protection Agency (EPA) air quality standards.

**Results:**

County-level analysis revealed a significant correlation (R = 0.68, R^2^ = 0.48, *p* < 0.001) between PM2.5 levels and overall stroke mortality. For every 1 μg/m^3^ increase in PM2.5, there was an increase of 1.89 ischemic stroke deaths per 100,000 residents. Racial and socioeconomic disparities were evident. Counties with predominantly Black populations exhibited a stark disparity, with each 1 μg/m^3^ increase in PM2.5 correlating with a significant rise in mortality, amounting to 5.81 additional deaths per 100,000 residents. Persistently poor counties displayed vulnerability, experiencing a 4.05 increase in ischemic stroke deaths per 100,000 residents for every 1 μg/m^3^ increase in PM2.5 levels. Conversely, in counties with a White majority and counties without a persistent state of poverty, the associated increases in stroke mortality per 100,000 residents for every 1 μg/m^3^ rise in county-level PM2.5 were 1.85 and 1.60, respectively. Counties with a majority of Black residents were over twice as likely to be non-compliant with EPA air quality standards compared to predominantly White counties (aOR 2.36 95% CI: 1.27–4.38, *p* = 0.006).

**Conclusion:**

This study underscores the significant impact of county-level air pollution, particularly PM2.5, on ischemic stroke mortality among older U.S. residents. Our findings indicate that counties with predominantly Black populations and those experiencing persistent poverty not only suffer from higher mortality rates but also are more likely to be non-compliant with EPA air quality standards. Targeted interventions and policies are urgently needed to reduce air pollution in these vulnerable communities and promote equitable public health outcomes.

## Introduction

Air pollution is a firmly established risk factor for cardiovascular diseases, with extensive research highlighting the adverse effects of PM2.5, a common air pollutant, on cardiovascular health (1–4). PM2.5, defined as particulate matter with a diameter of 2.5 micrometers or smaller, consists of minute solid particles and liquid droplets suspended in the air. Globally, PM2.5 is acknowledged as a major contributor to ischemic stroke (2–5). Despite the well-documented link between air pollution and stroke, the precise mechanisms underlying PM2.5-induced strokes are intricate and multifaceted.

The current prevailing mechanisms by which PM2.5 contributes to stroke risk involves a cascade of events. PM2.5, composed of tiny particles and chemical compounds, can be inhaled deeply into the respiratory system. Within the body, PM2.5 triggers oxidative stress and inflammation, promotes endothelial dysfunction, and elevates blood pressure. These pathological processes collectively increase the susceptibility to atherosclerosis, arterial plaque formation, and ultimately, ischemic stroke (5).

Furthermore, studies have consistently identified troubling disparities in PM2.5 exposure, with racial and ethnic minorities and poor residents experiencing higher levels of exposure (6, 7). This inequity in exposure has been linked to a corresponding disparity in PM2.5-attributable cardiovascular deaths, disproportionately affecting these vulnerable populations (1). Importantly, some studies have raised concerns that policies aimed at reducing air pollution may not effectively target marginalized populations, potentially exacerbating existing disparities in exposure and health outcomes (8).

Despite the existing knowledge on air pollution’s adverse health effects and disparities in exposure, there is a notable research gap. Few studies have systematically examined the interplay of race, socioeconomic status, county-level PM2.5 exposure, and stroke mortality in older residents. This study seeks to address this critical gap by focusing on the impact of county-level air pollution, specifically PM2.5, on ischemic and hemorrhagic stroke mortality among individuals aged 65 and older in the United States. It places a special emphasis on unraveling the relationship between race, socioeconomic status, education, and stroke mortality, filling a void in the current scientific understanding of these complex interactions.

## Methods

We did not pursue Institutional Review Board (IRB) approval for this study as we used publicly available datasets devoid of any personally identifiable information. Our research aligns with the guidelines outlined in the Strengthening the Reporting of Observational Studies in Epidemiology (STROBE) recommendations (9).

### Data sources

We collected county-level data on stroke mortality among adults aged 65 and older from the Center for Disease Control (CDC) WONDER database and Interactive Atlas on Heart Disease and Stroke, spanning the years 2016–2020 (10). Our analysis excluded counties in Puerto Rico (*n* = 77), American Samoa (*n* = 1), Guam (*n* = 1), and the U.S. Virgin Islands (*n* = 3). This dataset included annual mortality rates for both ischemic and hemorrhagic stroke in adults 65 years and older, presented per 100,000 individuals.

Furthermore, we gathered county-level information related to stroke risk factors. This encompassed data on the prevalence of specific health conditions among residents, including coronary artery disease (CAD), hypertension (HTN), Diabetes Mellitus (DM), obesity, high cholesterol (HLD), and smoking habits. Additionally, we collected data on the average annual levels of PM2.5 in each county.

To identify persistently impoverished counties, we referred to the Census Bureau’s American Community Survey data, which includes county level demographic data obtained from county profiles (11, 12). This information included details on racial and ethnic composition, total population figures, poverty rates, median household income, rural or urban designation and income inequality as measured by the Gini (G) index. A Gini coefficient ranges from 0 to 1. 0 indicates perfect equality where the income is equally split and a coefficient of 1 indicates perfect inequality, in which only one group or individual receives all the income. The reported racial/ethnic groups in the Census Bureau reports encompassed non-Hispanic White, non-Hispanic Black, Asian, Native Hawaiian and Other Pacific Islander, American Indian and Alaska Native, and Hispanic populations.

### Defining predictors

Our study delved into the racial discrepancies in the effects of county-level PM2.5 concentrations on ischemic and hemorrhagic stroke mortality by comparing predominantly non-Hispanic White, non-Hispanic Black, and Hispanic counties. A majority status for a racial or ethnic group was ascertained when it surpassed 50% of the county’s population. Socioeconomic disparities were analyzed by contrasting PM2.5’s impact on stroke mortality in counties with persistent poverty versus those without. Counties were recognized as persistently poor if they exhibited poverty rates of 20% or more for a continuous stretch of 30 years, a condition linked to systemic hurdles such as inadequate access to healthcare, education, and affordable nutrition. Further, we investigated the role of nationwide educational attainment in moderating the link between yearly PM2.5 levels and stroke mortality rates. For this purpose, we categorized counties into two groups based on educational attainment: those with a higher educational level and those with a lower one. This division hinged on the percentage of the population aged 21 and above lacking a college degree, with counties above the median percentage of 77.30% deemed lower educated and those below as higher educated. We further categorized counties based on their PM2.5 exposure levels using the current National Ambient Air Quality Standards (NAAQS) set by the Environmental Protection Agency (EPA). Counties were classified into two groups: those with ‘low’ levels of PM2.5, defined as annual ambient concentrations of 9.0 μg/m^3^ or less, and those with ‘high’ levels, which exceeded this threshold (13).

### Statistical analysis

We reported baseline descriptive characteristics for the study cohort which used t-tests for comparison of continuous variables. Our analysis began with univariable linear regression using PM2.5 levels as predictors for ischemic and hemorrhagic stroke. Subsequently, we conducted a multivariable multiple regression analysis to predict ischemic and hemorrhagic stroke mortality rates, incorporating the following variables: county-level PM2.5 concentrations in μg/m^3^ of air, county-level prevalence of stroke risk factors (CAD, HTN, DM, HLD, obesity, and smoking,) along with median household income, poverty rate, and Gini index. Additionally, we included the percentage of the county population without health insurance and the percentage of the county residents living within half a mile of a park. In all our multivariable linear regression analysis, the equation used to predict stroke mortality is represented as follows: Stroke Mortality = β₀ + (β₁ × PM2.5) + (β₂ × CAD) + (β₃ × HTN) + (β₄ × HLD) + (β₅ × DM) + (β₆ × Obesity) + (β₇ × Smoking) + (β₈ × Income) + (β₉ × Gini) + (β₁₀ × Poverty)  + (β₁₁ × Insurance) + (β₁₂ × Park Access).Unstandardized coefficients for the impact of PM2.5 on stroke mortality were compared among county level racial predictor groups, between persistently and non-persistently poor counties, and between higher and lower educated counties. Univariable logistic regression models were first employed to evaluate the likelihood of counties having high levels of PM2.5 as defined by the NAAQS. These models were subsequently expanded into multivariable logistic regression analyses that incorporated several covariates, including poverty rate, median household income, Gini index, rural or urban designation, state and park access. All tests for significance were two-sided, with a *p*-value less than or equal to 0.05 considered statistically significant. Analyses were performed using IBM SPSS Statistics (version 4.0.2).

## Results

**Figure 1:**
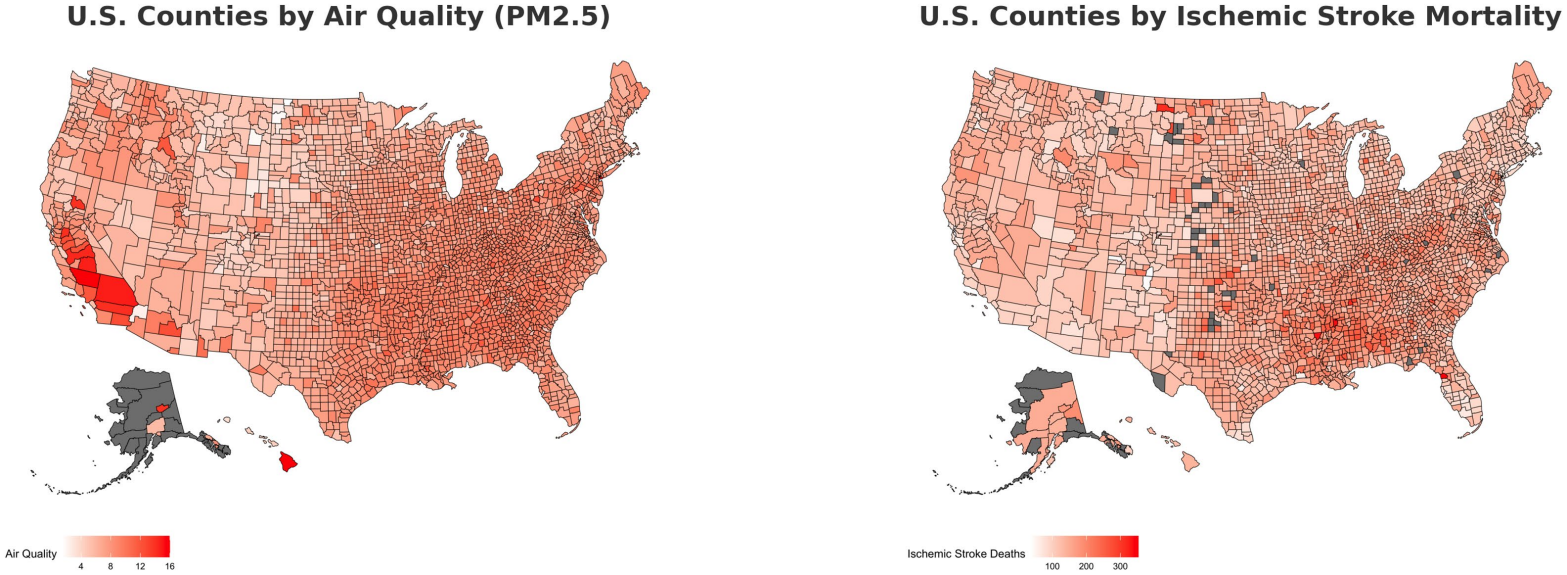
Heat map of air quality and stroke mortality.

This study covered a total 3,142 counties and county equivalents in the United States. Within the race/ethnicity sample (*n* = 2,917), 2,773 were predominantly White, 53 were predominantly Black, and 91 were predominantly Hispanic. In the socioeconomic sample (*n* = 3,142), 2,813 counties had no persistent poverty and 329 counties had persistent poverty. Within the persistent poverty subset, the majority (77.40%) were predominantly Black counties, starkly contrasted to predominantly White (8.50%) and predominantly Hispanic (7.90%) counties. In the education sample (*n* = 3,099), 1,185 counties had high educational attainment and 1,914 counties had low educational attainment. Figure 1 offers a visual representation of the relationship between ischemic stroke mortality and PM2.5 levels across U.S. counties throughout the duration of the study.

### County stroke risk factors

**Figure 2:**
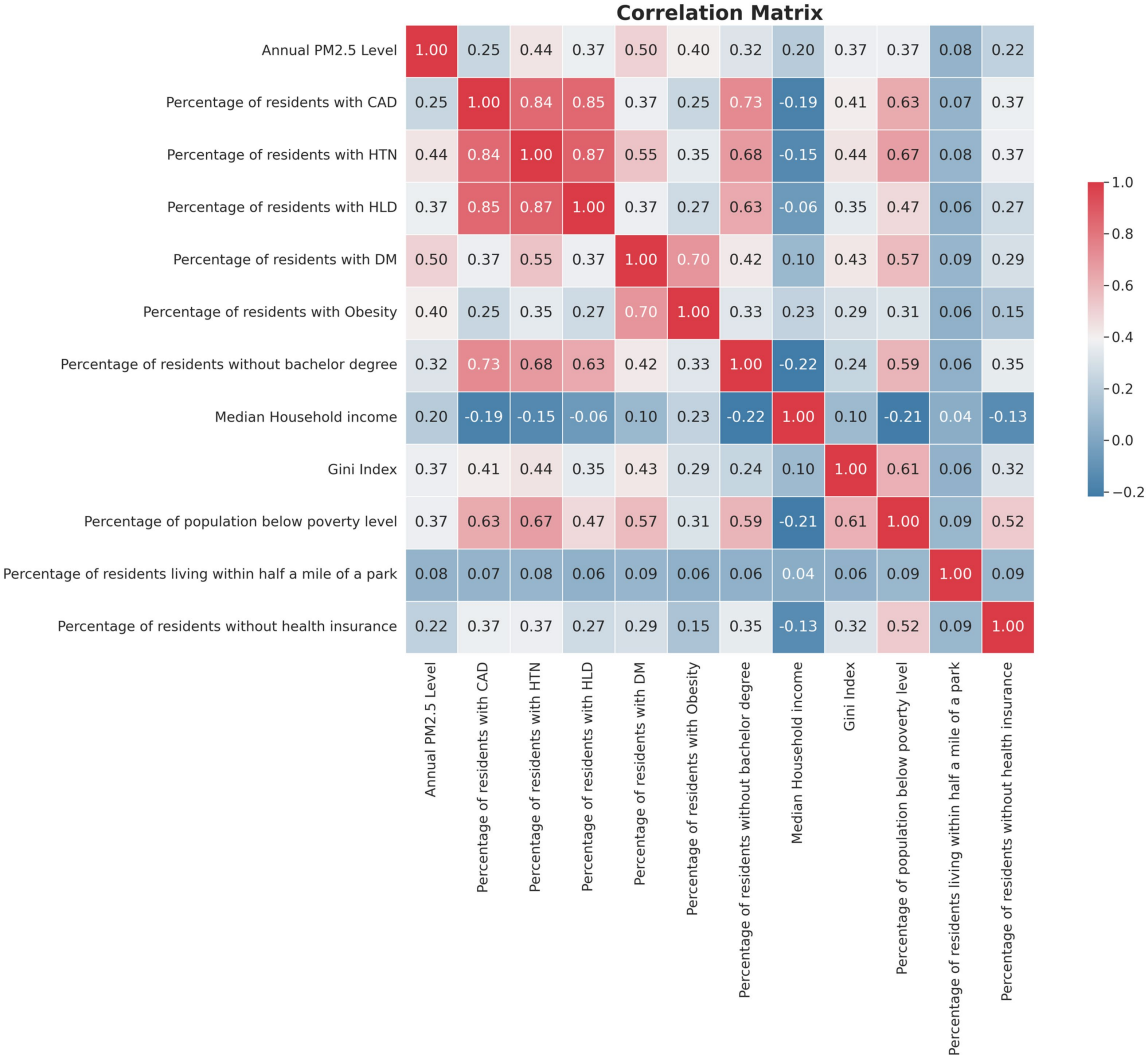
Correlation matrix of county-level pm2.5 and key variables in regression analysis.

The study further explored stroke risk factors at the county level, revealing notable differences based on racial/ethnic group predominance and persistent poverty. As detailed in Table 1, predominantly Black counties exhibited a significantly higher prevalence of all studied stroke risk factors compared to predominantly White counties. When comparing majority Hispanic to White counties, similar patterns were observed except in the case of obesity, where no statistically significant difference was found (28.49% in White-majority counties vs. 28.56% in Hispanic-majority counties, *p* = 1.000). In addition, counties classified as persistently poor showed a statistically higher prevalence of stroke risk factors than their non-persistently poor counterparts. These findings underscore the interplay between race, socioeconomic status, and the prevalence of health conditions that elevate stroke risk (Table 1). Figure 2 presents a comprehensive correlation matrix that delineates the interrelationships between county-level PM2.5 levels and key variables utilized in the regression analysis.

**Table 1:** Comparison by prevalence (%) of stroke risk factors by county demographics, poverty status, and educational attainment.

		Race/Ethnicity			Socioeconomic		Education	
Risk factor	Prevalence	White (*n* = 2,773)	Black (*n* = 53)	Hispanic (*n* = 91)	No Persistent Poverty (*n* = 2,813)	Persistent poverty (*n* = 29)	High (*n* = 1,185)	Low (*n* = 1914)
CAD	M	8.17	9.93	9.05	8.06	9.45	6.98	8.96
	SD	1.54	1.45	1.47	1.50	1.68	1.27	1.24
	*p-value*	Ref.	**<0.001**	**<0.001**	**<0.001**		**<0.001**	
HTN	M	37.00	44.87	39.88	36.44	43.23	33.03	39.70
	SD	5.56	6.16	5.59	5.14	6.89	4.37	4.97
	*p-value*	Ref.	**<0.001**	**<0.001**	**<0.001**		**<0.001**	
HLD	M	35.19	38.33	36.20	35.00	37.04	32.87	36.66
	SD	3.40	3.40	3.40	3.31	4.15	3.12	2.83
	*p-value*	Ref.	**<0.001**	**0.016**	**<0.001**		**<0.001**	
DM	M	8.70	10.10	9.28	8.55	10.34	8.02	9.19
	SD	1.59	2.02	1.70	1.45	2.03	1.30	1.64
	*p-value*	Ref.	**<0.001**	**0.002**	**<0.001**		**<0.001**	
Obesity	M	28.49	30.86	28.56	28.26	30.84	26.96	29.50
	SD	4.79	6.20	4.96	4.60	5.94	4.30	4.88
	*p-value*	Ref.	**0.001**	1.000	**<0.001**		**<0.001**	
Smoking	M	18.79	24.09	21.11	18.43	23.15	15.71	20.92
	SD	3.83	3.91	4.11	3.59	4.46	2.51	3.37
	*p-value*	Ref.	**<0.001**	**<0.001**	**<0.001**		**<0.001**	

CAD, coronary artery disease; HTN, Hypertension; HLD, Hyperlipidemia; DM, Diabetes Mellitus; M, Mean; SD, Standard Deviation. Bold values in table represent statistically significant comparisons.

**Figure 3:**
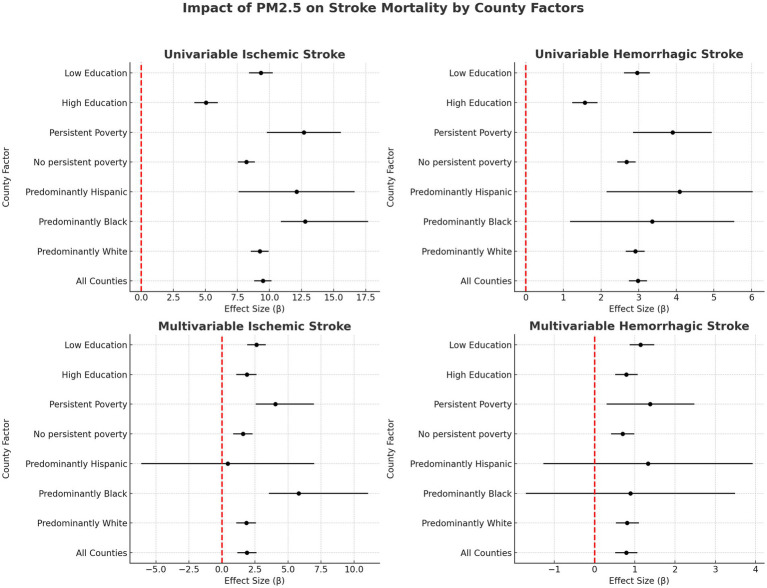
Impact of PM2.5 on stroke mortality by county factors.

### Stroke mortality and PM2.5 levels

Over the study period, the average annual mortality rate from ischemic stroke among older Americans was 143.50 per 100,000 across all counties, while hemorrhagic strokes had an average mortality rate of 52.78 per 100,000. The mean county-level PM2.5 concentration was 7.64 μg/m^3^.

The study highlighted significant racial and ethnic disparities in stroke mortality. Counties with predominantly White populations had lower ischemic stroke mortality rates (143.03 per 100,000) compared to predominantly Black (179.39 per 100,000, *p* < 0.001) and Hispanic-majority counties (152.97 per 100,000, *p* = 0.025). A similar trend was observed for hemorrhagic stroke mortality, with lower rates in predominantly White counties (52.66 per 100,000) than in Black-majority counties (64.15 per 100,000, *p* < 0.001). Counties with a majority Black population also had higher average annual PM2.5 levels (8.47 μg/m^3^) than predominantly White counties (7.63 μg/m^3^, *p* < 0.001). However, PM2.5 levels did not significantly differ between Hispanic-majority and White-majority counties (7.63 μg/m^3^ vs. 7.92 μg/m^3^, *p* < 0.344).

Comparatively, persistently poor counties experienced higher annual mortality rates for both ischemic and hemorrhagic strokes per 100,000 individuals (140.01 vs. 173.50, *p* < 0.001 and 51.92 vs. 60.09, *p* < 0.001, respectively) in unadjusted models. These counties also recorded higher annual PM2.5 levels (8.60 μg/m^3^ vs. 7.53 μg/m^3^, *p* < 0.001).

In counties with low educational attainment, higher mortality rates were found for both ischemic (155.04 vs. 125.13, *p* < 0.001) and hemorrhagic strokes (56.39 vs. 47.21, *p* < 0.001) per 100,000 residents, compared to those with high educational attainment. Counties with lower educational levels also had higher PM2.5 concentrations (8.06 μg/m^3^ vs. 6.96 μg/m^3^, *p* < 0.001).

The attributable risk of PM2.5 exposure on ischemic stroke mortality varied across racial groups and socioeconomic conditions. In predominantly White counties, the attributable risk stood at 13.89 deaths per 100,000 residents, resulting in approximately 27,951 deaths per year. For predominantly Black and Hispanic counties, the risks were 49.21 and 5.86 deaths per 100,000 residents, resulting in about 7,199 and 1,013 deaths per year, respectively.

For hemorrhagic stroke, the attributable risks were 6.18, 7.54, and 11.09 deaths per 100,000 residents for White, Black, and Hispanic counties, respectively, translating to approximately 12,440, 1,103, and 1,916 deaths per year.

In persistently poor counties, the attributable risk for ischemic stroke was 34.49 deaths per 100,000 residents (approximately 12,294 deaths per year), compared to 11.90 in non-persistent poverty counties (about 4,241 deaths per year). For hemorrhagic stroke, the risks were 11.78 and 5.27 deaths per 100,000 residents, resulting in roughly 4,200 and 1,879 deaths per year.

In lower-educated counties, the attributable risk for ischemic stroke was 9.26 deaths per 100,000 residents (around 12,777 deaths per year), compared to 5.50 in higher-educated counties (approximately 6,485 deaths per year). For hemorrhagic stroke, the risks were 21.04 and 13.22 deaths per 100,000 residents, resulting in approximately 29,023 and 15,597 deaths per year, respectively.

### Correlation between PM2.5 and county-level stroke mortality

In assessing the relationship between PM2.5 levels and stroke mortality, our univariable regression analysis revealed a moderate and statistically significant correlation for ischemic stroke (R = 0.45, *p* < 0.001, R^2^ = 0.20), with each 1 μg/m^3^ increase in PM2.5 levels corresponding to an increase of 9.48 deaths per 100,000 residents. Hemorrhagic stroke also showed a significant correlation (R = 0.41, *p* < 0.001, R^2^ = 0.17), with a slightly lower impact rate of 2.98 additional deaths per 100,000 residents for each 1 μg/m^3^ increase in PM2.5. The multivariable regression models further reinforced these findings, showing a strong correlation between PM2.5 levels and ischemic stroke mortality (R = 0.68, *p* < 0.0011, R^2^ = 0.46, β = 1.89) and a significant, albeit weaker, correlation for hemorrhagic stroke (R = 0.60, *p* < 0.001, R^2^ = 0.36, β = 0.79). Table 2, provides a comprehensive overview of the strength of the regression models, while Figure 3 illustrates the specific association between PM2.5 exposure and stroke mortality. The results, detailed in Figure 3, emphasize a stronger association with ischemic stroke, underscoring the heightened impact of PM2.5 on this particular type of stroke.

**Table 2:** Linear regression R and R^2^ values by county race/ethnicity, poverty, and education level.

	Univariable						Multivariable					
	Ischemic stroke			Hemorrhagic stroke			Ischemic stroke			Hemorrhagic stroke		
	R	R^2^	*p*	R	R^2^	*p*	R	R^2^	*p*	R	R^2^	*p*
Demographics												
All counties	0.45	0.20	**<0.001**	0.41	0.17	<0.001	0.68	0.46	**<0.001**	0.60	0.36	**<0.001**
Race/Ethnicity												
White	0.45	0.20	**<0.001**	0.41	0.17	**<0.001**	0.67	0.45	**<0.001**	0.59	0.35	**<0.001**
Black	0.47	0.23	**<0.001**	0.32	0.10	**0.003**	0.72	0.51	**<0.001**	0.56	0.31	**0.001**
Hispanic	0.50	0.25	**<0.001**	0.43	0.18	**<0.001**	0.67	0.45	**<0.001**	0.69	0.48	**<0.001**
Socioeconomic												
No persistent poverty	0.42	0.18	**<0.001**	0.39	0.15	**<0.001**	0.64	0.41	**<0.001**	0.57	0.32	**<0.001**
Persistent poverty	0.44	0.19	**<0.001**	0.39	0.15	**<0.001**	0.70	0.49	**<0.001**	0.63	0.40	**<0.001**
Education												
High	0.30	0.09	**<0.001**	0.26	0.07	**<0.001**	0.57	0.32	**<0.001**	0.48	0.23	**<0.001**
Low	0.41	0.17	**<0.001**	0.37	0.14	**<0.001**	0.62	0.38	**<0.001**	0.54	0.29	**<0.001**

Bold values in table represent statistically significant comparisons.

### Correlation between PM2.5 and stroke mortality by county predominant race/ethnicity

In univariate regression, an increase in PM2.5 was associated with a notably higher increase in annual ischemic stroke mortality in predominantly Black counties (β = 12.79, 95%CI 10.89–17.69, *p* < 0.001) compared to predominantly White counties (β = 9.24, 95%CI 8.55–9.94, *p* < 0.031). This disparity persisted in the multivariable regression analysis, indicating a significantly greater impact of PM2.5 on ischemic stroke mortality in predominantly Black counties (β = 5.81, 95%CI 3.55–11.07, *p* = 0.031) compared to White-majority counties (β = 1.82, 95%CI 1.08–2.58, *p* < 0.001). However, no statistically significant differences were noted in the influence of PM2.5 on county-level hemorrhagic stroke mortality between majority White and Black counties in either univariate or multivariable regression. Additionally, the analysis did not reveal significant differences in the correlation between PM2.5 levels and ischemic or hemorrhagic stroke mortality in predominantly Hispanic compared to White-majority counties. These findings, summarized in Figure 3, highlight the racial disparities in the health impact of air pollution.

### Correlation between PM2.5 and stroke mortality by persistent county poverty status

In univariable regression, persistently poor counties (β = 12.67, 95%CI 9.79–15.56, *p* < 0.001) were associated with a 4.48 deaths per 100,000 residents higher increase in annual mortality for every 1 μg/m^3^ increase in PM2.5 compared to counties that have not experienced persistent poverty (β = 8.19, 95%CI 7.53–8.86, *p* < 0.001). While the difference between the increase in annual county-level stroke mortality due to elevated air pollution decreased in multivariable regression, persistently poor counties (β = 4.01, 95%CI 2.55–6.90, *p =* 0.007) still had a higher correlated mortality compared to non-persistently poor counties (β = 1.58, 95%CI 0.86–2.32, *p* < 0.001). No statistically significant difference was found between county-level persistent poverty status and PM2.5-associated hemorrhagic stroke mortality in univariate or multivariate regression (Figure 3).

### Subgroup analysis of race, persistent poverty, and PM2.5 exposure on stroke mortality

In unadjusted regression analyses, predominantly Black and persistently poor counties experienced an increase of 11.73 ischemic stroke deaths per 100,000 residents for every 1 μg/m^3^ increase in PM2.5 (95% CI 5.59–17.87, *p* < 0.001). This compares to an increase of 10.92 deaths per 100,000 residents in predominantly White and persistently poor counties (95% CI 8.52–13.31, *p* < 0.001). However, multivariable analysis revealed a smaller and statistically non-significant increase of 7.93 deaths per 100,000 residents in predominantly Black and persistently poor counties (95% CI -1.31 to 17.12, *p* = 0.90) and an increase of 2.55 deaths per 100,000 residents in predominantly White and persistently poor counties (95% CI -0.03 to 5.13, *p* = 0.53). For hemorrhagic stroke, persistently poor and predominantly Black counties were associated with an increase of 3.53 deaths per 100,000 residents (95% CI, 1.34–5.71; *p* = 0.002), compared to an increase of 3.28 deaths per 100,000 residents (95% CI, 2.19–4.37, *p* < 0.001) in predominantly White and persistently poor counties. However, no statistically significant differences were observed in the adjusted models.

### Correlation between PM2.5 and stroke mortality by county level educational attainment

In our unadjusted models, we found a significant disparity in ischemic stroke mortality rates associated with county-level PM2.5 exposure, based on educational attainment. Specifically, counties with lower educational attainment demonstrated a more pronounced increase in ischemic stroke mortality (β = 9.32, 95%CI 8.40–10.25, *p* < 0.001) compared to their higher-educated counterparts (β = 5.05, 95%CI 4.14–5.97, *p* < 0.001). A similar pattern emerged for hemorrhagic stroke mortality, with low-education counties experiencing a greater increase (β = 2.96, 95%CI 2.61–3.30, *p* < 0.001) compared to high-education counties (β = 1.57, 95%CI 1.23–1.91, *p* < 0.001).

However, when we examined multivariable models, the association did not remain statistically significant for hemorrhagic stroke mortality in low-education counties (β = 1.14, 95%CI 0.87–1.42, *p* < 0.001) versus high-education counties (β = 0.79, 95%CI 0.510–1.07, *p <* 0.001), similarly the difference was not statistically significant for ischemic stroke mortality between low-education (β = 2.61, 95%CI 1.91–3.32, *p* < 0.001) and high-education counties (β = 1.90, 95%CI 1.18–2.61, *p <* 0.001).

### Assessment of county compliance with EPA NAAQS for PM2.5

Compared to predominantly White counties, those with a majority of Black residents were more than twice as likely to be non-compliant with EPA air quality standards, with an odds ratio (OR) of 2.36 (95% CI: 1.33–4.20, *p* = 0.004). However, no significant difference was observed between predominantly White and Hispanic counties (OR: 0.76, 95% CI: 0.42–1.39, *p* = 0.377). These patterns remained consistent in multivariate analyses, where the likelihood of non-compliance for predominantly Black versus White counties was 2.36 (95% CI: 1.27–4.38, *p* = 0.006), and for predominantly Hispanic versus White counties, it was 0.77 (95% CI: 0.41–1.44, *p* = 0.413).Counties with persistent poverty were nearly four times as likely to exceed EPA-recommended PM2.5 levels, with an OR of 3.95 (95% CI: 3.10–5.03, *p* < 0.001). This association remained significant even after adjusting for potential confounders [aOR: 1.67 (95% CI: 1.24–2.24, *p* < 0.001)].Educational level within counties showed no statistically significant association with compliance to NAAQS guidelines for air quality (OR: 0.94, 95% CI: 0.78–1.13, *p* = 0.511), a finding that was stable even after adjustments (aOR: 0.95, 95% CI: 0.76–1.17, *p* = 0.603).

## Discussion

While PM2.5 exposure has negative impacts on all counties, our research unveils significant disparities in its associated impact on ischemic stroke mortality, predominantly affecting older residents in Black-majority and economically disadvantaged counties in the United States. These disparities are entrenched in historical and socio-economic contexts, where minority and low-income communities are more likely to be situated near sources of pollution due to longstanding patterns of residential segregation and industrial zoning (14, 15). This proximity increases their exposure to harmful air pollutants like PM2.5. Notably, our study found a stronger correlation between PM2.5 levels and ischemic stroke mortality compared to hemorrhagic stroke mortality, aligning with current scientific literature and the mechanistic pathways of PM2.5 exposure detailed in the introduction (5, 16). These communities, burdened by limited healthcare access and a higher prevalence of comorbidities, are doubly impacted. The convergence of heightened pollution exposure and existing health vulnerabilities leads to a pronounced increase in stroke mortality, illustrating a multifaceted public health challenge. Furthermore, the higher rates of stroke mortality observed in the Black population may be influenced by a combination of biological, environmental, and socioeconomic factors. Genetic predispositions, higher prevalence of hypertension and diabetes, and greater exposure to environmental pollutants in predominantly Black residential areas can exacerbate the adverse health effects of PM2.5. Disparities in healthcare access, preventive services, and socioeconomic challenges further compound these risks.

Strikingly, our study indicates a correlation between counties with an above-average number of resident’s lacking bachelor’s degrees and higher rates of ischemic and hemorrhagic stroke mortality. This relationship persisted after adjusting for confounders, but the difference remained statistically significant for hemorrhagic stroke mortality. The inverse relationship between educational attainment and cardiovascular disease is well understood and can be attributed to lower health literacy as well as increased risk factors such as smoking, obesity, and physical inactivity (17, 18). Our findings highlight how the association between stroke mortality and air pollution is complicated by educational attainment and exacerbated by socioeconomic disparities.

While counties with a majority of Black residents and those characterized by persistent poverty were shown to have heightened non-compliance with EPA air quality standards, it is the confluence of socioeconomic adversity, rather than race alone, that appears to amplify these disparities. This observation is evident in our subgroup analysis, which shows that racial disparities in stroke mortality related to PM2.5 exposure diminish when comparing persistently poor counties with majority Black populations to those with majority White populations. This indicates that socioeconomic status may serve as a fundamental axis along which health disparities, as they pertain to air quality, manifest. Such conclusions draw parallels with research from metropolitan contexts like New York City, where socioeconomic disparities, compounded by differences in healthcare access, emerge as salient determinants of the health inequities tied to environmental pollution (19).These findings are consistent with the weathering hypothesis, which posits that chronic exposure to social and economic disadvantage has cumulative negative effects on health, thus exacerbating disparities along racial lines when combined with socioeconomic factors (20–23). Therefore, interventions aimed at mitigating PM2.5 levels and their effects should not only be environmentally focused but also socioeconomically targeted to address the root causes of these public health challenges.

In areas predominantly inhabited by Black populations, those marked by persistent poverty, and regions with lower educational attainment, the intersection of environmental and social factors forms a complex matrix of health disparities. These marginalized communities often grapple with systemic challenges like inadequate public health funding and limited educational resources (24). This not only restricts their access to information about the health risks associated with air pollution but also impedes their ability to advocate for improved environmental and healthcare conditions (5–8, 14–18, 24–26). Consequently, a self-perpetuating cycle emerges where racial, socioeconomic, educational marginalization is intertwined with poorer health outcomes and increased environmental exposure. These factors are compounded by under-resourced healthcare systems and limited economic opportunities, exacerbating the impact of air pollution.

The stark disparities revealed in our study indicate a broader issue of environmental injustice. This phenomenon, where environmental risks disproportionately plague minority and low-income communities, is rooted in systemic and structural inequalities, like segregation, economic disparity, and political marginalization; which further increase the burden of pollution in these communities (14, 15, 25, 26). These marginalized communities often lack sufficient green spaces, which are essential for mitigating pollution and enhancing health benefits (27, 28). Lacking green spaces not only reduces quality of life but also intensifies the urban heat island effect, thereby amplifying the detrimental effects of pollutants like PM2.5 (29).

Compounding this scenario is the looming threat of climate change, which is poised to exacerbate existing environmental hazards (30). Climate change, manifesting in more frequent and severe heatwaves, can increase pollution levels and disproportionately affect marginalized communities (31–33). Marginalized communities, often lacking the resources to adapt to rapidly changing environmental conditions, are vulnerable to the worsening impacts of climate change (32–35). This intersection of environmental injustice and climate change necessitates urgent and comprehensive strategies that address the immediate environmental challenges and the underlying socio-economic inequities.

Addressing the disparities in our study requires a proactive, multifaceted approach. Policy interventions are crucial and should prioritize strengthening and enforcing air quality regulations, especially in disadvantaged communities. These policies must be equitable, ensuring that the benefits of improved air quality are experienced by all (8). The goal should be to move beyond mere regulatory compliance to actively work towards narrowing the disparities in air pollution exposure. Community involvement in environmental decision-making is critical for the effectiveness of these policies. Empowering affected communities through grassroots movements and initiatives can lead to more inclusive and responsive environmental policies. Ensuring that the voices and concerns of those most impacted by pollution are heard and addressed is key to developing sustainable and equitable solutions.

In tandem with policy interventions, enhancing access to healthcare and education in vulnerable communities is imperative. Improved healthcare access can enable better management of pollution-related health issues and associated comorbidities, ultimately reducing the health burden of air pollution. Educational initiatives play a vital role as well, raising awareness about the risks of air pollution and fostering preventive measures. Such efforts can empower individuals and communities to advocate for healthier environments. Investment in infrastructure improvements is also a critical component of addressing these disparities. Developing green spaces and enhancing public transportation in disadvantaged areas can significantly reduce pollution exposure. These improvements not only contribute to better air quality but also improve the overall livability and health of these communities.

### Limitations

Though insightful, our study has several limitations inherent to its design and data sources: chiefly, the reliance on secondary, publicly available datasets. While these datasets scope broadly, they lack granularity regarding individual health specifics and precise environmental exposures; lacking personal identifiers precluded a more nuanced analysis within subpopulations. Additionally, the cross-sectional nature of the data hindered our ability to establish causality between PM2.5 exposure and stroke mortality. Furthermore, dichotomizing the socioeconomic variables prevented us from capturing the intricate socioeconomic dynamics within counties, risking an oversimplified interpretation of the complex interplay between socioeconomic factors and health outcomes. Another limitation surfaced with our reliance on county-level averages, potentially masking important within-county variations and inviting an ecological fallacy if findings are extrapolated to individuals. Despite employing robust regression methods, residual confounding due to unaccounted factors such as variations in healthcare access or local pollution sources remains possible. A significant limitation is the lack of individual-level income information, which results in unaddressable individual-level confounding. This limitation prevents us from determining whether the higher rates of ischemic and hemorrhagic stroke mortality among Black populations are solely due to a higher percentage of low-income individuals within this group. Future research should explore this by examining the risks among low-income counties to minimize confounding by socioeconomic status, thereby assessing if poor White Americans face similar risks as poor Black Americans per μg/m^3^ of PM2.5 exposure. Additionally, a similar stratified analysis should be conducted for populations living in areas with varying levels of education to gain further insights into what is driving the risk differences that we currently attribute to race. To address these limitations and enhance our understanding of the issues uncovered, future research should focus on longitudinal studies that can trace the impact of PM2.5 exposure on individual health outcomes and establish causality. Lastly, it is crucial for future researchers to explore interventions that mitigate the disparities revealed in this study to develop more effective and targeted public health policies.

## Conclusion

This study highlights the impact of PM2.5 air pollution on ischemic stroke mortality among Older Americans. It underscores the presence of troubling disparities in PM2.5 exposure, with racial/ethnic minorities and persistently impoverished counties experiencing higher levels of air pollution. Moreover, the study reveals the differential impact of PM2.5 on ischemic stroke mortality in Older Americans, emphasizing the need for tailored prevention and intervention strategies. Addressing these disparities in exposure and health outcomes is essential for achieving health equity and reducing the burden of stroke in vulnerable populations. Comprehensive efforts, including regulatory policies to reduce air pollution and promote equitable access to clean air, are imperative to mitigate the adverse effects of air pollution.

## Data Availability

Publicly available datasets were analyzed in this study. This data can be found at: 1. https://www.cdc.gov/dhdsp/maps/atlas/index.htm, 2. https://www.census.gov/programs-surveys/acs, 3. https://wonder.cdc.gov, and 4. https://www.census.gov/acs/www/data/data-tables-and-tools/data-profiles/.
